# A randomised, assessor blind, parallel group comparative efficacy trial of three products for the treatment of head lice in children - melaleuca oil and lavender oil, pyrethrins and piperonyl butoxide, and a "suffocation" product

**DOI:** 10.1186/1471-5945-10-6

**Published:** 2010-08-20

**Authors:** Stephen C Barker, Phillip M Altman

**Affiliations:** 1 Parasitology Section, School of Chemistry & Molecular Biosciences, and UniQuest Pty. Ltd., University of Queensland, St Lucia, Queensland, 4072 Australia; 2 Altman Biomedical Consulting Pty. Ltd., 20 Folly Point, Cammeray, New South Wales, Australia

## Abstract

**Background:**

There are many different types of pediculicides available OTC in Australia. In this study we compare the efficacy and safety of three topical pediculicides: a pediculicide containing melaleuca oil (tea tree oil) and lavender oil (TTO/LO); a head lice "suffocation" product; and a product containing pyrethrins and piperonyl butoxide (P/PB).

**Method:**

This study was a randomised, assessor-blind, comparative, parallel study of 123 subjects with live head lice. The head lice products were applied according to the manufacturer's instructions (the TTO/LO product and the "suffocation" product were applied three times at weekly intervals according to manufacturers instructions (on Day 0, Day 7 and Day 14) and the P/PB product was applied twice according to manufacturers instructions (on Day 0 and Day 7)). The presence or absence of live lice one day following the last treatment was determined.

**Results:**

The percentage of subjects who were louse-free one day after the last treatment with the product containing tea tree oil and lavender oil (41/42; 97.6%) and the head lice "suffocation" product (40/41, 97.6%) was significantly higher compared to the percentage of subjects who were louse-free one day after the last treatment with the product containing pyrethrins and piperonyl butoxide (10/40, 25.0%; adj. p < 0.0001).

**Conclusion:**

The high efficacy of the TTO/LO product and the head lice "suffocation" product offers an alternative to the pyrethrins-based product.

**Trial Registration:**

The study was entered into the Australian/New Zealand Clinical Trial Registry, ACTRN12610000179033.

## Background

The incidence of head louse infestation is high in many countries[1-3]. This may be explained, in part at least, by the evolution in head lice of lower susceptibility (resistance) to older pediculicides[4]. Two new types of head lice products have found wide acceptance in many countries: essential oil based products and products designed to "suffocate" head lice. It is important to assess and compare the lice kill rates and the safety of these newer products with existing market leading products in well controlled and well designed clinical trials.

One essential oil based product containing 11.0% eucalyptus oil was reported to have an efficacy of 82.5% compared to a kill rate of 36.1% for a product containing pyrethrins and piperonyl butoxide using a study design similar to that employed in this study[5]. In another study a "suffocation" product containing 5% benzyl alcohol was reported to kill all head lice in 92.2% of subjects as measured one day after the second treatment (day 8)[6]. Using the same head lice product containing pyrethrins and piperonyl butoxide, studied by ourselves previously, as a comparator[5], the efficacy and safety of two new head lice products were studied by us: NeutraLice Lotion^® ^(containing melaleuca oil and lavender oil) and NeutraLice Advance^® ^(a "suffocation" product).

## Methods

### Objectives and Interventions

To compare the efficacy and tolerance of three head lice treatment products when used according to the manufacturers instructions:

1. Product containing melaleuca oil (tea tree oil) 10% w/v and lavender oil 1% w/v (TTO/LO) (NeutraLice Lotion^® ^Key Pharmaceuticals Pty Ltd, Australia) presented as a clear oily solution

2. "suffocation" product containing benzyl alcohol, mineral oil, polysorbate 80, sorbitan monooleate, Carbopol 934, water and triethanolamine (NeutraLice Advance^® ^Key Pharmaceuticals Pty Ltd, Australia) presented as a white opaque lotion

3. Product containing pyrethrins 1.65 mg/g, and piperonyl butoxide 16.5 mg/g (P/PB) (Banlice Mousse^® ^Johnson & Johnson Pacific Pty Ltd, Australia) presented as a pressurised aerosol mousse

### Methodology

This was an assessor blind, randomised, parallel group, comparative study. The study population consisted of primary school-aged children (aged 4 yrs to 12 yrs) from three different schools in Queensland and their siblings with live head lice (adults or nymphs) in their hair or on their scalp. If parental written informed consent was provided, the children were screened for the presence of live head lice by visual inspection (see Appendix 1 - Definitions) and by dry-combing (see Appendix 1 - Definitions). Those subjects meeting the entry criteria were randomised and treated with one of the three head lice products. Subjects with a history of allergies, presence of scalp disease and those who were treated with a head lice product in the 4 weeks prior to participation in this trial were excluded.

The TTO/LO and "suffocation" products were applied three times, at weekly intervals, as per the manufacturer's instructions (on day 0, day 7 and day 14). The P/PB product was applied twice, as per the manufacturer's instructions (on day 0 and day 7). The louse-combing procedure normally used in combination with these three products was not done so we could compare the efficacy of the components of each product without confounding the efficacy measurements by physically removing head lice by combing (which is a treatment in itself).

The primary outcome measure was the louse free rate (see Appendix 1 - Definitions) assessed one day after the last treatment (at day 15 for TTO/LO and "suffocation" products and at day 8 after application of P/PB product) and was determined by wet-combing (see Appendix 1 - Definitions) for the Intention to Treat population and the Per Protocol population. A secondary outcome measure, louse free rate at day 1, was determined by dry-combing

#### Ethics

This trial was conducted in compliance with the World Medical Association Declaration of Helsinki, the requirements of the National Statement on Ethical Conduct in Research Involving Humans, ICH E6 Guidance for the Industry; Good Clinical Practice: Consolidated Guidance, the National Privacy Principles and relevant State/Territory laws. The trial activities were approved by the Medical Research Ethics Committee of the University of Queensland, project No. 2003000184 and all parents/guardians provided written informed consent.

#### Randomisation

Eligible subjects were randomly assigned to receive one of the three head lice treatments by a computer generated code using blocked randomisation (groups of six).

#### Blinding

This trial was assessor-blind. The person applying the treatment could not avoid being aware of the product being applied as the products are easily identifiable by their physical attributes; however, assessor-blinding was achieved by using different staff for applications on the one hand and assessment and CRF data entry on the other hand, and by physically separating these activities at the investigational site. Subjects were prevented from sighting the products being used. The parents of subjects were also blinded to the treatment applications. Analysts involved in data management were blinded to the identification of each treatment group until the final efficacy analysis for each treatment group was complete.

#### Treatment of siblings

If an enrolled subject had a primary-school aged sibling (aged 4 years to 12 years), the sibling was also examined for head lice and, if infested with live lice and available for enrolment, this sibling was enrolled into the same treatment arm as the subject. Those siblings not enrolled, because they were unavailable for the trial for any reason or had eggs only, were wet-combed-out at days 0 and 7 for siblings of subjects receiving the P/PB product or wet-combed-out (see Appendix 1 - Definitions) at days 0, 7 and 14 for siblings of subjects receiving TTO/LO and "suffocation" products.

#### Treatment compliance

The *Intent-to-Treat *(ITT) population is defined as all subjects receiving at least one treatment application. This was the primary population for determination of safety and efficacy.

Subjects who met all the protocol requirements are termed the *per-protocol *(PP) population and this was the secondary population for determination of efficacy. Subjects are considered to be *per protocol *(PP) if they satisfied the requirements listed in Appendix 2:

#### Dosage and dosage regimen

The doses, method of application and number of weekly treatments were those recommended by the manufacturers. All three products were applied for 10 minutes. After the TTO/LO product was applied the hair was covered by a shower cap made of polyvinyl chloride to retain the volatile components of the formulation. The TTO/LO and "suffocation" products were then washed out with water; the P/PB product was washed out with a standard shampoo. The TTO/LO and "suffocation" products were applied at weekly intervals on days 0, 7 and 14. The P/PB product was applied on days 0 and 7.

#### Criteria for evaluation of efficacy

The efficacy of each product is defined as the "louse-free rate." The louse free rate was assessed at day 1 (by dry-combing), and at day 15 (by wet-combing) for those subjects treated with TTO/LO or "suffocation" products OR at day 8 (by wet-combing) for those subjects treated with the P/PB product. In the case of the day 1 examination, dry-combing was used and combing was stopped immediately if live lice were observed. A blinded assessor conducted the hair and scalp examinations.

Often, visual inspection was sufficient to determine if live lice are present especially in cases of severe infestation. In such cases, dry/wet-combing was not necessary to confirm the presence of live lice. However, visual inspection alone was insufficient to declare a subject as "louse-free".

#### Criteria for evaluation of safety (tolerance)

Subjects were interviewed on site regarding possible adverse effects during and immediately following the application procedure as well as just before the next scheduled application by site staff. The incidence and severity of adverse events was compared between treatment groups. The ITT population was analysed for safety.

#### Statistical and data management methods

##### Determination of sample size and data analysis

Previous efficacy studies involving the P/PB product reported a cure rate of 36.1%[4]. Assuming the efficacy of the TTO/LO and "suffocation" products to be approximately 70% in the ITT population, it was estimated that 40 subjects in each group (assuming clustering, i.e. siblings will receive the same treatment as the first subject enrolled in the family) were required to test the hypothesis of superiority of the TTO/LO and "suffocation" products with a two-sided test using alpha at or less than 0.025 to allow for two pair-wise comparisons with 75% power. For the unadjusted analysis the chi-square test was used. For adjusted analysis the Generalized Estimating Equations methodology was used to fit the logistic regression model to account for the clustering within families.

## Results

### Efficacy

**Figure 1:**
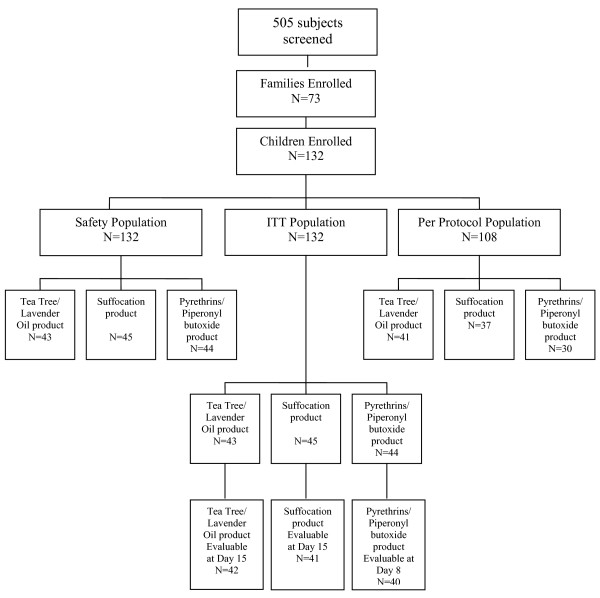
**Disposition of subjects**.

Subjects were enrolled between April and June 2009. The disposition of subjects is shown in Figure 1. 505 subjects were screened, 132 were enrolled in the study (43 were treated with TTO/LO product, 45 with "suffocation" product and 44 with the P/PB product). Of these 132 subjects (ITT population), 123 subjects were evaluable: 42 TTO/LO subjects; 41 "suffocation" product subjects and 40 P/PB subjects; 9 subjects were not assessed at a final visit. Of the 132 enrolled subjects, 108 were deemed PP; 41 TTO/LO subjects, 37 "suffocation" product subjects and 30 P/PB subjects. Reasons for a subject being deemed not PP were: enrolled sibling not treated with the same product as original subject; use of an alternative head lice treatment during the trial; siblings not available for treatment on same day as original subject; and subjects not available for assessment. There were no subject withdrawals. The day after the last treatment was day 15 for the TTO/LO and "suffocation" products and day 8 for the P/PB product.

With regard to the primary efficacy endpoint for the ITT population (123 subjects in total who were assessed after the final treatment) (Table 1), subjects in the TTO/LO product group were more likely to be louse-free on the day after the last treatment than subjects in the P/PB product group (41 out of 42 subjects, 97.6% vs. 10 out of 40 subjects, 25.0%; adj. p < 0.0001). In addition, subjects in the "suffocation" product group were more likely to be louse-free than subjects in the P/PB product group (40 out of 41 subjects, 97.6% vs. 10 out of 40 subjects, 25.0%; adj. p < 0.0001). An analysis in which the nine subjects who were not assessed after the final treatment, when treated as if they were non-responders, yielded similar results (Table 1).

**Table 1:** Louse-Free Rate the Day After Final Treatment (ITT Population)

				P-value	
		**Number Louse-free**	**Louse-free Percentage**	**Unadjusted**	**Adjusted**

Louse-free rate: Tea Tree/Lavender Oil product versus Pyrethrins/piperonyl butoxide product	Tea Tree/Lavender Oil product	41 out of 42 (41 out of 43)	97.62% (95.35%)	< 0.0001	< 0.0001
	
	Pyrethrins/piperonyl butoxide product	10 out of 40 (10 out of 44)	25.0% (22.73%)		

Louse-free rate: Suffocation product versus Pyrethrins/piperonyl butoxide product	Suffocation product	40 out of 41 (40 out of 45)	97.56% (88.89%)	< 0.0001	< 0.0001
	
	Pyrethrins/piperonyl butoxide product	10 out of 40 (10 out of 44)	25.0% (22.73%)		

Subjects in the ITT population for whom there was no information regarding whether or not they had lice on the day after the last treatment (9 subjects) were excluded from the first analysis of 123 subjects. When these 9 subjects were included in the analysis and counted as failures (132 subjects), the results are shown in brackets.

With regard to the secondary endpoint of louse-free rate for the PP population (108 subjects in total) (Table 2) one day following final treatment, subjects in the PP population in the TTO/LO product group were more likely to be louse-free on the day after the final treatment than subjects in the P/PB product group (40 out of 41 subjects, 97.6% vs. 10 out of 30 subjects, 33.3%; unadj. p < 0.0001). Similarly, subjects in the PP population in the "suffocation" product group were more likely to be louse-free on the day after the final treatment than subjects in the P/PB product group (37 out of 37 subjects, 100.0% vs. 10 out of 30 subjects, 33.3%; unadj. p < 0.0001).

**Table 2:** Louse-free Rate the Day After Final Treatment (PP population)

				P-value	
		**Number Louse-free**	**Louse-free Percentage**	**Unadjusted**	Adjusted*

Louse-free rate: Tea Tree/Lavender Oil product versus Pyrethrins/piperonyl butoxide product	Tea Tree/Lavender Oil product	40 out of 41	97.56	< 0.0001	*
	
	Pyrethrins/piperonyl butoxide product	10 out of 30	33.33		

Louse-free rate: Suffocation product versus Pyrethrins/piperonyl butoxide product	Suffocation product	37 out of 37	100	< 0.0001	*
	
	Pyrethrins/piperonyl butoxide product	10 out of 30	33.33		

*The adjusted p-values could not be calculated because the generalized Hessian matrix was not positive definitive (the off-diagonal elements are too few)

With regard to the louse-free rates on day 1 (one day after the first application for all treatments, secondary endpoint), subjects in the ITT population in the TTO/LO product group were more likely to be louse-free on day 1 than subjects in the P/PB product group (90.3% vs. 43.3%; adj. p = 0.001). Similarly, subjects in the ITT population in the "suffocation" product group were more likely to be louse-free on day 1 than subjects in the P/PB product group (69.4% vs. 43.3%; adj. p = 0.0329). For this population on day 1, the difference in louse-free rates for TTO/LO and "suffocation" products was not statistically significant (p = 0.1009). Subjects in the PP population in the TTO/LO product group were more likely to be louse-free on day 1 compared to subjects in the P/PB product group (90.0% vs. 57.1%; adj. p = 0.0196). For the PP population on day 1, 72.4% of the subjects in the "suffocation" product group were louse-free, whereas 57.1% of the subjects in the P/PB product group were louse-free; this difference was not statistically significant (adj. p = 0.2986).

Demographic factors were not found to be confounders of the effect of treatment on outcome since they were not associated with the outcome. Using a multiple logistic regression model adjusting for gender, hair length, hair type, school attended and accounting for clustering, there was still a statistically significant higher louse-free rate at the day after the final treatment among children treated with the TTO/LO product (p-value = < 0.0001) and among children treated with the "suffocation" product (p-value = < 0.0001) than among children treated with the P/PB product.

### Safety (tolerance)

Of the 132 subjects enrolled, 36 adverse events were reported; 29 in relation to TTO/LO treatment; 3 in relation to "suffocation" product; and, 4 were related to P/PB product treatment. All adverse events reported were considered by the investigator to be related to study treatment. No adverse reaction was considered to be serious by definition. Some subjects reported more than one adverse event - 20 TTO/LO product-subjects (46.5%), 3 "suffocation" product-subjects (6.7%) and 4 P/PB product-subjects (9.1%) reported at least one adverse event.

The adverse events for the TTO/LO, "suffocation" and P/PB products were rated as mild in severity with the exception of 3 adverse events in relation to the TTO/LO product which were rated as moderate in intensity. These 3 adverse events occurred in 3 subjects and were described as stinging of the eyes following product contact with the eyes; stinging of the neck; and, erythema of the skin. In one case following product-contact (TTO/LO) with the eyes, the application was washed out prior to the 10 minute contact time. In all other cases where an adverse event was reported no action was taken or required.

The most commonly reported adverse events in relation to the TTO/LO product were stinging (13 subjects, 30.2%), flaky scalp/dry scalp (8 subjects, 18.6%) and erythema (4 subjects, 9.3%). In the case of the "suffocation" product 3 subjects (6.7%) reported flaky scalp/dry scalp. In the case of the P/PB product, 3 subjects (6.8%) reported flaky scalp/dry scalp and 1 subject (2.3%) reported erythema.

The reported stinging or burning sensation associated with the TTO/LO product (which has been reported before with other essential oil based head louse products[5]) lasted from 3 minutes to 141 minutes and erythema was reported to last from 5 minutes to 185 minutes in various subjects. Only one case of erythema was reported with the P/PB product and its duration was not recorded. Flaky scalp/dry scalp, when it occurred in relation to the TTO/LO, "suffocation" or P/PB products appeared to last for at least one day.

## Discussion

The efficacy of the P/PB product in this study was similar to that reported for the same product in a previous and similarly designed study of ours (25% as compared to 36.1%, respectively)[5]. The efficacy of the TTO/LO product (97.6%), however, exceeded the efficacy of another essential oil product studied by us in a similarly designed study (Moov Head Lice Solution^®^, 82.5%)[5]. The efficacy of the "suffocation" product we tested was 97.6% compared to 92.2% for another "suffocation" product, which also contains 5% benzyl alcohol[6]. The mechanism of action of essential oils in the treatment of head lice is unknown but "suffocation" products are thought to act by blocking the "breathing" spiracles of lice. It has been postulated that benzyl alcohol may contribute to the efficacy of suffocating products by "stunning" the spiracles open and allowing the product to block the respiratory apparatus.

It is likely, that the use of a shower cap to trap volatile components of essential oils such as melaleuca oil and eucalyptus oil contributes to the higher efficacy of these products compared to the same products applied without a shower cap. The use of a shower cap with essential oil products, however, also appears to be correlated to a higher incidence of transient mild to moderate stinging sensations, burning sensations, and erythema. The sensitivity of the skin of children varies. The "suffocation" product, which is highly efficacious yet caused little skin irritation in the present study, would be a good choice for children with inherently sensitive skin.

Wet combing is a very accurate method to diagnose active head lice infestation[7]. In contrast to previous studies by us, we determined efficacy one day after the last treatment rather than seven days after the last treatment, to reduce re-infestation, which, of course, confounds the assessment of efficacy[8]. In order to determine and compare the safety and efficacy of the products as they are used by parents and children, the products were applied strictly in accordance with the manufacturer's instructions. In the case of the P/PB product there were two applications, one week apart. In the case of the TTO/LO and "suffocation" products there were three applications one week apart. While some might criticise our study design, this study design allowed us to assess the efficacy of these products according to the way the products will be used by parents and children; and thus is highly desirable in our opinion. It is yet to be determined if head lice will readily develop resistance to essential oil products, which contain a large number of different active ingredients, and "suffocation" products, which do not act on the nervous system of the louse.

## Conclusions

The TTO/LO product and head lice "suffocation" product were both > 97% effective and were almost four times as effective as the P/PB product that we compared them with, when used according to manufacturer instructions. These results support the view that this new "suffocation" product is as effective in controlling head lice as an essential oil product applied with a shower cap.

## Competing interests

Associate Prof. Steve Barker and Dr. Phillip Altman have previously provided consultant-advice to Key Pharmaceuticals Pty. Ltd. the company that funded this study. The company had no active role in study design, study management, data analysis, interpretation of results or manuscript writing.

## Authors' contributions

PA and SB were responsible for study design. PA was responsible for protocol and document drafting, site staff training, study management, study monitoring, and writing the manuscript. SB conducted the trial and helped write the manuscript. Both read and approved the final manuscript.

## Appendix 1: Definitions

### • louse-free rate

The proportion of subjects on whom no live head lice (adults or nymphs) were found when combed by a specified method at a specified time point.

### • visual inspection

Visual inspection involves a brief examination of the hair assisted by parting of the hair in spots to determine whether live lice are present. It may be used in conjunction with dry-combing to determine active infestation with live lice.

### • dry-combing

Combing from scalp to the hair tips using a head lice comb and not using water or conditioner to assist combing. Every part of the hair should be combed with a metal-toothed lice comb up to 6 times. The hair may be de-tangled with a wide-gap comb before dry-combing.

### • wet-combing

Standard commercial conditioner is applied liberally to the hair, and the hair detangled with a regular wide-toothed comb. The hair is then combed with a metal-toothed lice comb to remove live lice. Every part of the hair is combed 6 times, scalp to hair tips. During combing, the comb is wiped onto a white tissue and the wipings are examined for lice. After combing, the conditioner is rinsed or towelled from the hair as desired by the subject. Wet-combing is a powerful detection technique to determine the final infestation status of a subject at the end of an efficacy trial, as the conditioner traps the lice making it highly unlikely that any lice in the hair will avoid detection. Thus, the likelihood that a subject will be incorrectly categorised as louse free when in fact a low grade infestation still exists is substantially reduced[7].

The wet-combing method results in the smothering of lice in conditioner and the removal of head lice from the scalp, thus wet-combing has an irreversible effect on a subject's infestation status. Wet-combing was only used at the completion of the head lice trial.

### • wet-combed-out

"Wet-combed-out" is a therapeutic procedure which uses the wet-combining technique (above) to ensure the hair is louse free: wet-combing is continued until no live lice are found in six continuous passes of the comb. This is repeated for every section of the hair.

## Appendix 2: Criteria for assessing a subject as Per Protocol

• They comply with all inclusion and exclusion protocol requirements

• Have a signed informed consent authorisation

• Treatment was administered on day 0, 7 and 14 for Tea Tree/Lavender Oil or suffocation products or treatment was administered on days 0 and 7 for the Pyrethrins/piperonyl butoxide product

• Are evaluated using the wet-combing procedure at one day after the final application (day 15 in the case of Tea Tree/Lavender Oil or suffocation products or day 8 in the case of Pyrethrins/piperonyl butoxide product)

• The subject's Case Report Form is sufficiently complete to enable a valid assessment of efficacy and safety.

• Have not used any other head lice products during the trial or in the 4 weeks preceding the trial

• Have not used any other head lice products other than those specified in the protocol during the trial

• Has not used a head lice comb during the trial

• Have not bleached or dyed their hair during the trial

• The subject's siblings were assessed and treated, if required, as per the section "Treatment of siblings"

## Pre-publication history

The pre-publication history for this paper can be accessed here:

http://www.biomedcentral.com/1471-5945/10/6/prepub
